# Placental inflammation in a fetal demise of a SARS-CoV-2-asymptomatic, COVID-19-unvaccinated pregnant woman: a case-report

**DOI:** 10.1186/s12884-024-06530-y

**Published:** 2024-04-25

**Authors:** Maricarmen Abrego-Navarro, Rodrigo Villalobos, Jaime Sanchez, Deisa Lamela, Cindy Fu, Erika Guerrero, Paulino Vigil-De Gracia, Sandra López-Vergès, Mairim A. Solis

**Affiliations:** 1 Stem Cell Research Group, Department of Research in Sexual and Reproductive Health Research, Gorgas Memorial Institute for Health Studies, Avenida Justo Arosemena y Calle 35, Panama City, Republic of Panama; 2 Present Address: Ministry of Health, Panama City, Republic of Panama; 3 Present Address: Department of Diagnostics, Pathology Service, Hospital Santo Tomas, Panama City, Republic of Panama; 4 Department of Gynecology and Obstetrics, Hospital Santo Tomas, Panama City, Republic of Panama; 5 Department of Research in Virology and Biotechnology, Gorgas Memorial Institute for Health Studies, Avenida Justo Arosemena y Calle 35, Panama City, Republic of Panama; 6 Division of Gynecology and Obstetrics, Complejo Hospitalario Metropolitano Dr Arnulfo Arias Madrid, Panama City, Republic of Panama; 7 Sistema Nacional de Investigación, SENACYT, Panama City, Republic of Panama

**Keywords:** Intrauterine fetal demise, SARS-CoV-2, COVID-19 in pregnancy, Placental pathology, Placental inflammation, Maternal vascular malperfusion, Fetal vascular malperfusion

## Abstract

**Background:**

Intrauterine fetal demise is a recognized complication of coronavirus disease 2019 in pregnant women and is associated with histopathological placental lesions. The pathological mechanism and virus-induced immune response in the placenta are not fully understood. A detailed description of severe acute respiratory syndrome coronavirus 2 (SARS-CoV-2)-induced inflammation in the placenta during fetal demise is crucial for improved clinical management.

**Case presentation:**

We report the case of a 27-week gestation SARS-CoV-2-asymptomatic unvaccinated pregnant woman without comorbidities or other risk factors for negative pregnancy outcomes with a diagnosis of intrauterine fetal demise. Histopathological findings corresponded to patterns of subacute inflammation throughout the anatomic compartments of the placenta, showing severe chorioamnionitis, chronic villitis and deciduitis, accompanied by maternal and fetal vascular malperfusion. Our immunohistochemistry results revealed infiltration of CD68^+^ macrophages, CD56^+^ Natural Killer cells and scarce CD8^+^ T cytotoxic lymphocytes at the site of placental inflammation, with the SARS-CoV-2 nucleocapsid located in stromal cells of the chorion and chorionic villi, and in decidual cells.

**Conclusion:**

This case describes novel histopathological lesions of inflammation with infiltration of plasma cells, neutrophils, macrophages, and natural killer cells associated with malperfusion in the placenta of a SARS-CoV-2-infected asymptomatic woman with intrauterine fetal demise. A better understanding of the inflammatory effects exerted by SARS-CoV-2 in the placenta will enable strategies for better clinical management of pregnant women unvaccinated for SARS-CoV-2 to avoid fatal fetal outcomes during future transmission waves.

## Background

Since the first cases of coronavirus disease 2019 (COVID-19) were reported in 2019, much has been learned about the effects of severe acute respiratory syndrome coronavirus 2 (SARS-CoV-2) infection during pregnancy. Although SARS-CoV-2 is rarely transmitted transplacentally to the fetus, histopathological alterations have been described in a subset of placentas from SARS-CoV-2-infected mothers [1]. These pathologies in the placenta have been associated with negative pregnancy outcomes, including preterm birth and intrauterine fetal demise [1–4]. The term SARS-CoV-2 placentitis describes SARS-CoV-2-infected placentas in cases of fetal demise, with three specific pathological lesions, chronic histiocytic intervillositis, massive perivillous fibrin deposition and trophoblast necrosis, which were rare before the COVID-19 pandemic [5]. The different pathological mechanisms and viral-induced immune responses in the placenta associated with SARS-CoV-2-related fetal demise are not fully understood. We report a novel histopathological lesion of inflammation with a description of the immune cells associated with the presence of SARS-CoV-2 in the placenta of a pregnant woman who was diagnosed with intrauterine fetal demise.

## Case presentation

A 20-year-old woman, gravida 3, para 2, presented with a pregnancy of 27 weeks to Hospital Santo Tomas with a 3-day history of absent fetal movements without other medical complaints. She had no significant past medical history or comorbidities for negative pregnancy outcomes. The maternal physical examination was unremarkable, and vital signs were within normal limits (Table 1). Obstetric sonography confirmed intrauterine fetal demise by revealing a 27-week fetus with an estimated body weight of 968 g and no fetal heart rate. There were no signs of placental abruption or preterm rupture of membranes. Maternal laboratory values were normal, and TORCH (toxoplasmosis, other agents, rubella, cytomegalovirus, and herpes simplex) infections were excluded (Table 1).

**Table 1:** Maternal and neonatal clinical characteristics of COVID-19-positive pregnant woman with fetal demise diagnosis. Hospital Santo Tomas, Panama 2020

**Maternal information**	
Date of stillbirth	September 2020
Maternal age (years)	20
Maternal history	Unremarkable
Gravidity	3
Parity	2
Gestational age (weeks)	27
Clinical severity	Asymptomatic
Type of delivery	Vaginal
**Physical exam**	
Temperature (°C)	36.8
Blood pressure (mmHg)	108/64
Heart rate (BPM)	81
Respiratory rate (CPM)	16
Oxygen saturation (%)	97
**Laboratory findings**	**Normal range**
Hemoglobin (g/dL)	11.9 (10.9–14.3)
Hematocrit (%)	35 (31.2–41.9)
Leukocytes (× 10^3/µL)	10.6 (3.8–11.8)
Neutrophils (× 10^3/µL)	3 (1.9–8.2)
Lymphocytes (× 10^3/µL)	2.4 (1.1–3.1)
Platelets (× 10^3/µL)	374 (179–408)
Fibrinogen (mg/dL)	492 (248–506)
**Virology and microbiology**	
Nasopharingeal SARS CoV-2 RT PCR	Positive
Syphillis	Negative
HIV	Negative
Other TORCH	Negative ^a^
**Ultrasound findings**	
Fetal growth	Normal
Fetal weight (g)	968
Amniotic fluid volume	Normal
Placental abruption	No sign
Preterm rupture of membranes	No sign
**Neonatal information**	**Normal range**
Sex	Male
Birthweight (g)	992
Birthweight percentile (%)	40 (10–90)
Fronto-occipital circumference (cm)	24
Fronto-occipital circumference percentile (%)	25 (10–90)
Crown-rump length (cm)	24
**Virology**	
SARS-CoV-2 RT‒PCR on fetus	Not done
SARS-CoV-2 RT‒PCR on placental tissue	Negative
**Fetal autopsy**	Not perfomed

^a^Patient declared negative for TORCH agents based on clinical history and hospital laboratory results during the data collection. *Abbreviations: BPM* Beats per minute, *COVID-19* Coronavirus disease 2019, *CPM* Cycles per minute, *HIV* Human immunodeficiency virus, *RT‒PCR* Reverse transcription polymerase chain reaction, *SARS-CoV-2* Severe acute respiratory syndrome coronavirus 2, *TORCH* Toxoplasmosis, other agents, rubella, cytomegalovirus, and herpes simplex

The mother was positive for SARS-CoV-2 through viral RNA nasopharyngeal swab analysis via reverse transcription polymerase chain reaction (RT–PCR) performed on all pregnant women before admission. She was admitted for labor induction via the use of Misoprostol. The patient delivered a male stillborn fetus of 992 g, corresponding to the 40th weight percentile, with a fronto-occipital circumference of 24 cm, equivalent to the 25th percentile for his gestational age, and a crown-rump length of 24 cm (Table 1). Macroscopic evaluation of the fetus did not show apparent morphological abnormalities. The patient was asymptomatic, and she was discharged without complications. The patient was not vaccinated against SARS-CoV-2 due to the unavailability of vaccines during the last quarter of 2020, when the major circulating variant in the country was the Panamanian endemic A.2.4. The National Bioethics Research Committee approved this study (EC-CNBI-2020–04-52), and written informed consent was obtained from the patient.

**Fig. 1:**
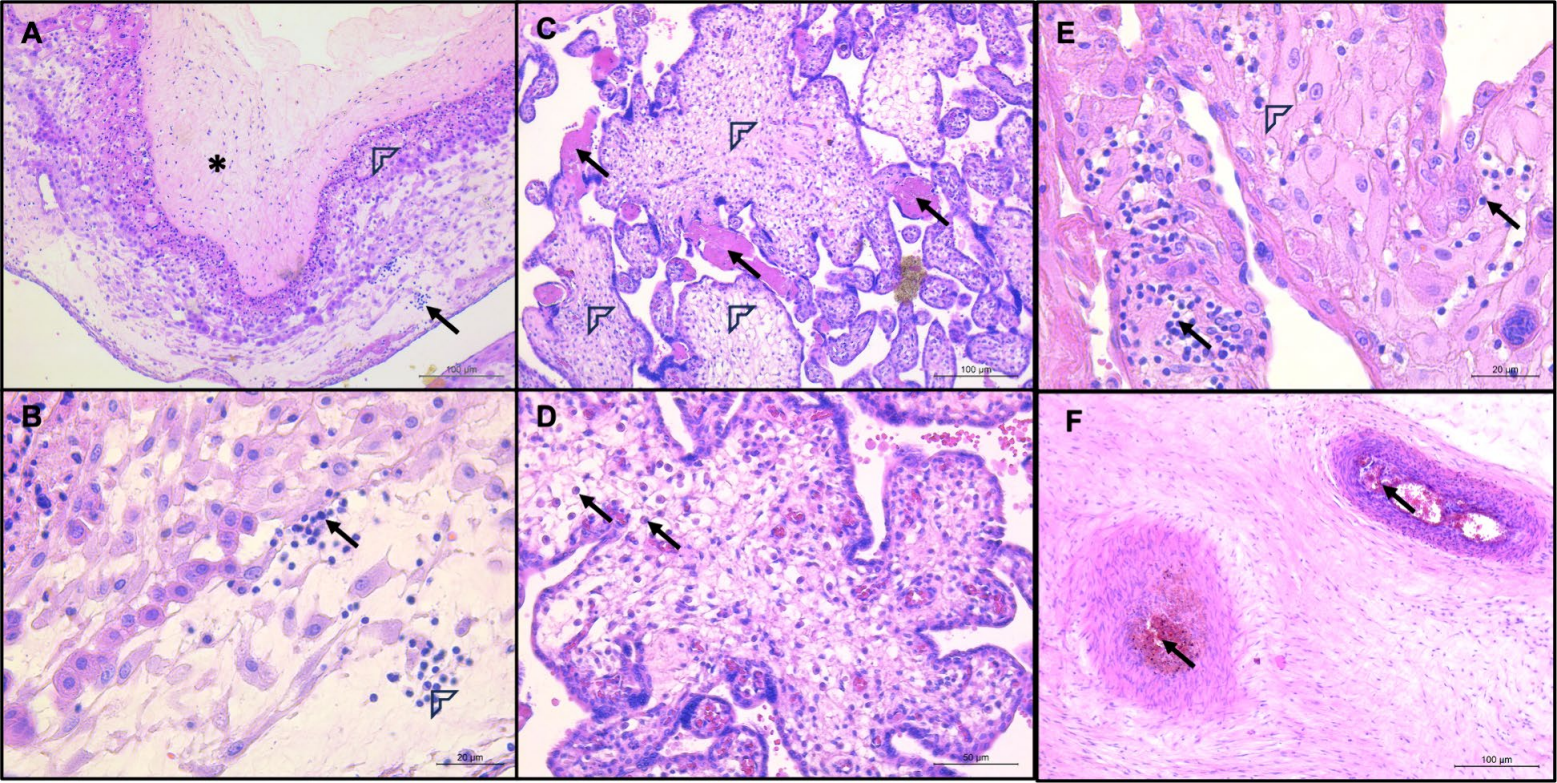
Histopathological findings in the placenta of a patient with fetal demise. **A** Severe chorioamnionitis shown in the chorion (asterisk), subchorionic fibrin (arrowhead) and decidua (arrow); H&E stain, 10 × magnification, scale bar: 100 µm; **B** Inflammatory infiltration in the fetal membranes, which consists of plasma cells (arrowhead) and lymphocytes (arrow); H&E stain, 40 × magnification; scale bar: 20 µm; **C,** Arrows show intervillous fibrin deposition and arrowheads, villitis; H&E stain, 10 × magnification, scale bar: 100 µm; **D** Villitis with infiltration of plasma cells (arrows), 20 × magnification; scale bar: 50 µm; **E** Infiltration of neutrophils (arrowhead) and lymphocytes (arrow) in the decidua basalis; H&E stain, 40 × magnification, scale bar: 20 µm; **F** Thrombi in stem villi (arrows); H&E stain, 10 × magnification, scale bar: 100 µm*.* Abbreviations: H&E: hematoxylin and eosin

The placenta was collected, and histopathological analysis was performed through hematoxylin and eosin staining by using diagnostic criteria from the Amsterdam Placental Workshop Group Consensus Statement. Severe chorioamnionitis was present throughout the chorion, subchorionic fibrin and decidua of the membranes (Fig. 1A), as was the identification of mononuclear cells that morphologically resembled plasma cells and lymphocytes within the fetal membranes (Fig. 1B). Maternal vascular malperfusion, which consisted of scarce intervillous fibrin deposition and accelerated villi maturation with the formation of a vasculo-syncytial membrane in chorionic villi, was observed (Fig. 1C). The chorionic villi showed inflammation with infiltrating plasma cells (Fig. 1D). The decidua basalis was necrotic with severe inflammation composed of lymphocytes and neutrophils (Fig. 1E). Stem villi with vessel obliteration and thrombi were observed, consistent with fetal vascular malperfusion (Fig. 1F).

**Fig. 2:**
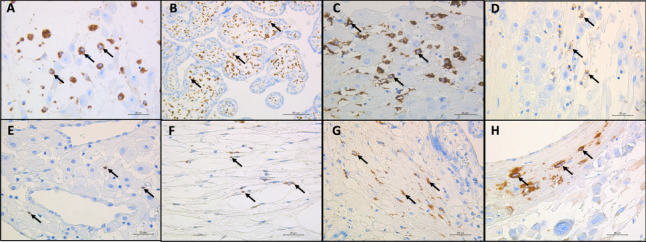
Immunohistochemistry of immune cell markers and the SARS-CoV-2 antigen in the placenta of a patient with fetal demise. **A** Macrophages detected at the interphase between the decidua of membranes and chorion (arrow); anti-CD68 immunohistochemical stain, 40 × magnification, scale bar: 20 µm; **B** Hyperplasia of macrophages in chorionic villi showed by arrows; anti-CD68 immunohistochemical stain, 20 × magnification, scale bar: 50 µm*;* **C,** Macrophages detected in the decidua represented by arrows; anti-CD68 immunohistochemical stain, 40 × magnification, scale bar: 20 µm; **D,** Natural killer cells in decidua basalis showed by arrows; anti-CD56 immunohistochemical stain, 40 × magnification, scale bar: 20 µm; **E** CD8 T cytotoxic lymphocytes detected in the decidua basalis represented by arrows; anti-CD8 immunohistochemical stain, 40 × magnification, scale bar: 20 µm; **F** SARS-COV2-infected stromal cells of the chorion represented by arrows; anti-SARS-CoV-2 nucleocapsid immunohistochemical stain, 40 × magnification, scale bar: 20 µm; **G**, SARS-CoV-2-infected stromal cells of chorionic villi represented by arrows; anti-SARS-CoV-2 nucleocapsid immunohistochemical stain, 40 × magnification, scale bar: 20 µm; **H** SARS-CoV-2-infected decidual cells represented by arrows; anti-SARS-CoV-2 nucleocapsid immunohistochemical stain, 40 × magnification, scale bar: 20 µm***.*** Abbreviations: SARS-CoV-2: *severe* acute respiratory syndrome coronavirus 2

The immunohistochemistry results demonstrated inflammation with infiltration of CD68^+^ macrophages at the chorionic plate (Fig. 2A), the chorionic villi (Fig. 2B) and the decidua (Fig. 2C) and of CD56^+^ natural killer (NK) cells (Fig. 2D) and scarce CD8^+^ cytotoxic T lymphocytes (Fig. 2E) in the decidua basalis. Placental tissue from the SARS-CoV-2-infected patient expressed the SARS-CoV-2 nucleocapsid (1:500, anti–SARS-CoV-2 nucleocapsid protein, Santa Cruz Biotechnologies, Santa Cruz, California) in stromal cells of the chorion (Fig. 2F), stromal cells of the chorionic villi (Fig. 2G), and decidual cells (Fig. 2H). SARS-CoV-2 viral RNA was not detected by RT‒PCR (Table 1) in the placental tissue.

**Fig. 3:**
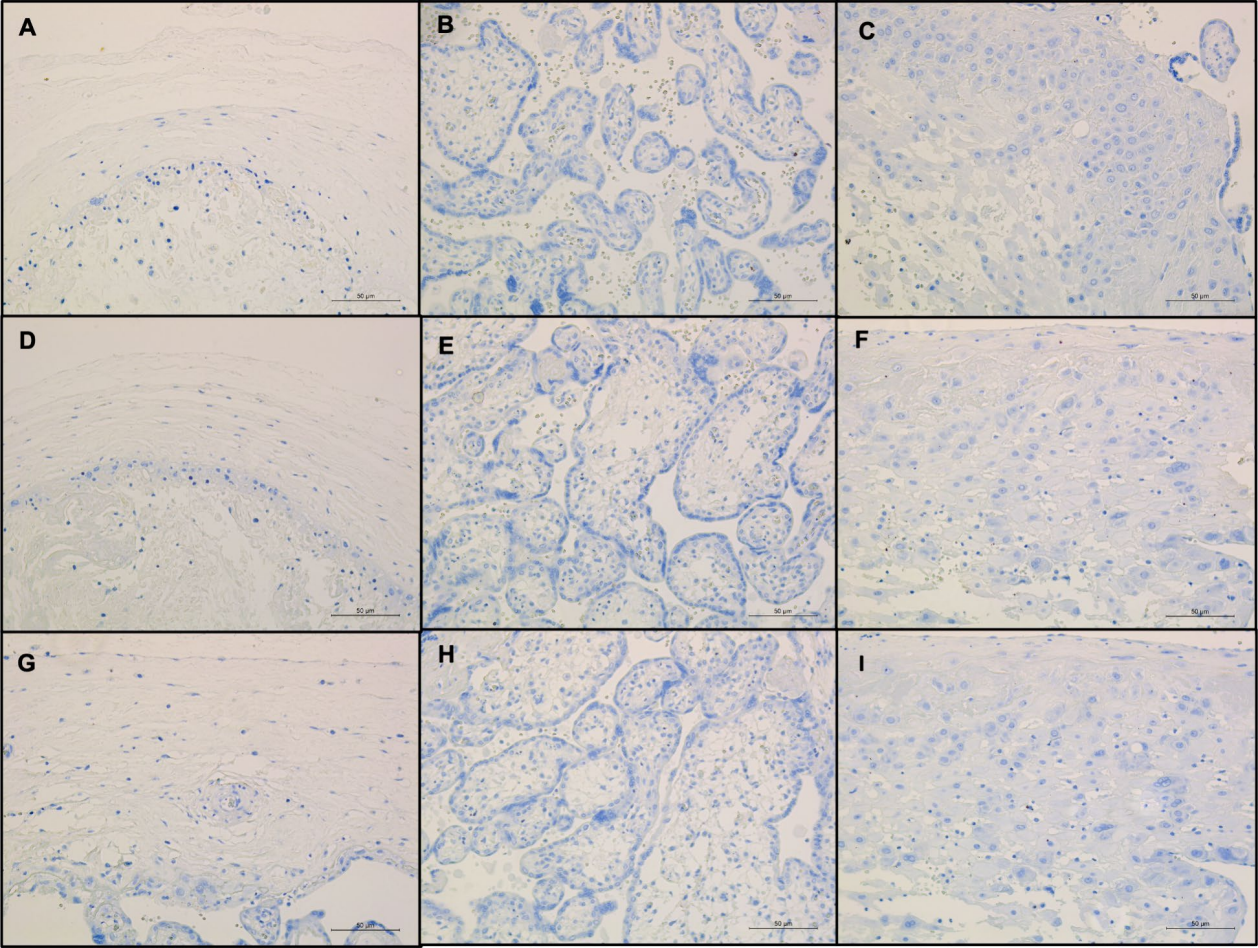
Immunohistochemical staining for CD19, CD4 and p53. **A-C** Chorion, chorionic villi and decidua basalis; anti-CD19 immunohistochemical stain, 20 × magnification; scale bar: 50 µm; **D-F** Chorion, chorionic villi and decidua basalis; anti-CD4 immunohistochemical stain, 20 × magnification; scale bar: 50 µm; **G-I** Chorion, chorionic villi and decidua basalis; anti-P53 immunohistochemical stain, 20 × magnification; scale bar: 50 µm

None of the compartments of the placenta stained positive for CD19^+^ B lymphocytes (Fig. 3A to C), CD4^+^ T lymphocytes (Fig. 3D to F), or p53^+^ apoptotic cells (Fig. 3G and I).

## Discussion and conclusion

Intrauterine fetal demise is a rare complication of COVID-19 in pregnant women and is associated with histopathological lesions induced by SARS-CoV-2 infection in the placenta. The pathological mechanism and virus-induced immune response in the placenta are not fully understood. We report the case of a 27-week gestation, SARS-CoV-2-infected asymptomatic woman with fetal demise. Studies have reported that SARS-CoV-2-associated fetal demise is not linked to the severity of the disease in pregnant mothers [6–8] and occurs between 21 and 39 weeks of gestation [7, 9], consistent with our case. Reports suggest that unvaccinated women with different clinical manifestations may develop placental pathological features with rapid deleterious fetal outcomes occurring between 3 and 15 days after receiving a COVID-19 diagnosis [10–12]. In our patient, maternal SARS-CoV-2 detection was followed by the diagnosis of fetal demise, due to asymptomatic status. This case arises before the emergence of the alpha and delta variants in Panama, which are linked to increased reports of fetal demise in SARS-CoV-2-infected mothers [7, 9].

The placental pathology present in cases of fetal demise in COVID-19 patients generally consists of three lesions: chronic histiocytic intervillositis, massive perivillous fibrin deposition [13, 14], and trophoblast necrosis; this triad is termed SARS-CoV-2 placentitis [15, 16]. It is suggested that SARS-CoV-2 placentitis may affect > 75% of the placenta [6], negatively compromising its function in gas and nutrient exchange and leading to severe fetal hypoxic-ischemic injury and fetal demise through malperfusion and placenta insufficiency [5, 9, 16]; many of these findings were rare before the COVID-19 pandemic. Our histopathological findings corresponded to patterns of subacute inflammation throughout the anatomic compartments of the placenta, showing severe chorioamnionitis, chronic villitis and deciduitis, accompanied by maternal and fetal vascular malperfusion with scarce intervillous fibrin deposition. In this patient, necrosis of trophoblasts, massive perivillous fibrin deposition, and histiocytic intervillositis were not observed, suggesting that other histopathological features in the placenta may be involved in the mechanisms that led to fetal demise. It is not clear whether the scarce fibrin deposition we observed was enough to compromise gas and nutrient exchange from the intervillous space to the chorionic villi; however, as we observed both maternal and fetal vascular malperfusion, we suggest that the sum of the findings played a role in the outcome of pregnancy.

Inflammation of the placental compartments is likely also crucial [8]. We identified CD68^+^ macrophages in the chorionic plate, chorionic villi, and decidua basalis. Other reports have identified macrophages in the intervillous space of the chorionic villi in SARS-CoV-2-associated fetal demises, consistent with one criterion of the SARS-CoV-2 placentitis triad [6, 14, 17, 18]. We additionally reported the presence of other immune cells, such as neutrophils, plasma cells, CD56 + NK cells and a few CD8^+^ T lymphocytes, which are implicated in antiviral responses, at sites of placental inflammation. Most cases reported the localization of SARS-CoV-2 spike and nucleocapsid antigens in syncytiotrophoblasts [9, 19], cytotrophoblasts [19], within villous stromal cells including Hofbauer cells and villous capillary endothelial cells [20, 21], and on the maternal side of the placenta [18, 21]. We detected the SARS-CoV-2 nucleocapsid in both maternal decidual cells and fetal stromal cells of the chorion and chorionic villi, consistent with the sites of inflammation.

Two possible mechanisms could be involved in fetal demise, one linked to placental vascular malperfusion and the other to an abnormal inflammatory immune response that leads to a cascade of fatal consequences. The fact that SARS-CoV-2 infection of the placenta and fetal demise are rare events but are consistently described in unvaccinated pregnant women demonstrates the importance of preventive measures in unvaccinated populations and highlights the urgency of vaccination in pregnant women, as we confirm that healthy patients can present SARS-CoV-2 placental lesions with fatal consequences.

## Data Availability

The datasets used and/or analyzed during the current study are available from the corresponding author upon reasonable request.

## Abbreviations

COVID-19: Coronavirus disease 2019
HIV: Human immunodeficiency virus
NK: Natural killer
RT‒PCR: Reverse transcription polymerase chain reaction
SARS-CoV-2: Severe acute respiratory syndrome coronavirus 2
TORCH: Toxoplasmosis, other agents, rubella, cytomegalovirus, and herpes simplex
